# Quality of life in employees exposed to organic solvents: A study of 196 cases

**DOI:** 10.1192/j.eurpsy.2023.671

**Published:** 2023-07-19

**Authors:** N. Ladhari, S. Chemingui, N. Mechergui, M. Mersni, S. Ernez, D. Brahim, H. Ben Said, G. Bahri, I. Youssef

**Affiliations:** Department of Occupational Medicine, Charles Nicolle Hospital of Tunis, Tunis, Tunisia

## Abstract

**Introduction:**

Occupational exposure to organic solvents remains a real risk for exposed employees, particularly in mental health and quality of life.

**Objectives:**

- To evaluate the quality of life of employees exposed to organic solvents

- To research the professional and extra-professional determinants of this quality of life.

**Methods:**

This is a descriptive cross-sectional study that compared 196 employees exposed to organic solvents with 64 non-exposed employees from the same socio-professional environment. The investigation took place in four different companies in the governorate of Tunis. An environmental study combining an evaluation of working conditions and atmospheric monitoring was carried out to identify and quantify exposure to solvents. Quality of life was assessed using the SF36 questionnaire in its Arabic version.

**Results:**

The solvent mixtures to which the employees were exposed mainly contained hexane, toluene, ethyl acetate, methyl ethyl ketone, cyclohexane, and perchloroethylene. Exposure to these solvents is primarily from glues and paint products. The study population was relatively young (34.1 years +/-9.8), predominantly male (sex ratio=2.2), with an education level of no more than secondary school in 90% of cases, with an average work experience of 10.3 years (+/-8.2) and represented mainly by manual workers (75.4%). The pathological history of the exposed patients was dominated by chronic neuropsychological disorders (48.1%). The global score of SF36 (SFG) was significantly poorer in the solvent-exposed group (SFG= 64.1+/- 21.1 versus 70.1+/-23.3) (p=0.05). Among the eight dimensions of the SF36, a very significant alteration of the dimensions: “perceived health ”, “psychological health” and “repercussion of psychological health on daily activities ” was noted in the solvent-exposed group.

The main determinants of the quality of life of workers exposed to solvents were: level of education, frequency of exposure, length of exposure, and company.

According to the job-exposure matrix, only “perceived health” appeared to be impaired by high levels of cumulative solvent exposure (p= 0.0006).

**Conclusions:**

According to this study, organic solvents can affect the quality of life of exposed employees by acting essentially on perceived health, psychological health, and the “impact of psychological health on daily activities”.

**Disclosure of Interest:**

None Declared

